# Bugs battle stress from hot blood

**DOI:** 10.7554/eLife.33035

**Published:** 2017-11-21

**Authors:** Joshua B Benoit, David L Denlinger

**Affiliations:** 1 Department of Biological SciencesUniversity of CincinnatiCincinnatiUnited States; 2 Department of EntomologyOhio State UniversityColumbusUnited States; 3 Department of Evolution, Ecology and Organismal BiologyOhio State UniversityColumbusUnited States

**Keywords:** thermoregulation, haematophagy, vector biology, functional morphology, insect physiology, *Rhodnius prolixus*, Other

## Abstract

A heat exchange mechanism in the head of kissing bugs helps to prevent stress and regulate their temperature while they feed on warm blood.

**Related research article** Lahondère C, Insausti TC, Paim RMM, Luan X, Belev G, Pereira MH, Ianowski JP, Lazzari CR. 2017. Countercurrent heat exchange and thermoregulation during blood-feeding in kissing bugs. *eLife* **6**:e26107. doi: 10.7554/eLife.26107

Over 14,000 species of insects, arachnids and other arthropods feed on the blood of vertebrates. This blood-feeding lifestyle appears to have evolved independently at least six times since the Jurassic and Cretaceous periods, and perhaps up to 20 times (Benoit et al., 2014; Lehane, 2005; Sterkel et al., 2017). Blood is notoriously devoid of many essential micronutrients, but it is a good source of the proteins and lipids that are essential for development and the generation of eggs in arthropods (Lehane, 2005; Coast, 2009; Sterkel et al., 2017).

Acquiring a blood meal is laden with considerable risks before, during and after feeding (Figure 1). For example, the sheer size difference between the vertebrate and the blood-feeder makes the defensive actions of the host potentially lethal for the arthropod, and so must be avoided. One strategy commonly used to avoid detection is for the arthropod to ingest large blood meals to minimize how often feeding needs to occur. Such gluttony can lead to dramatic increases in size: mosquitoes and tsetse flies grow 2–3 times bigger after a blood meal (Benoit et al., 2014; Lehane, 2005; Coast, 2009), whereas ticks and kissing bugs (which spread Chagas disease in South America and the southern United States) expand 10–100 fold (Sonenshine and Roe, 2014).

**Figure 1.**
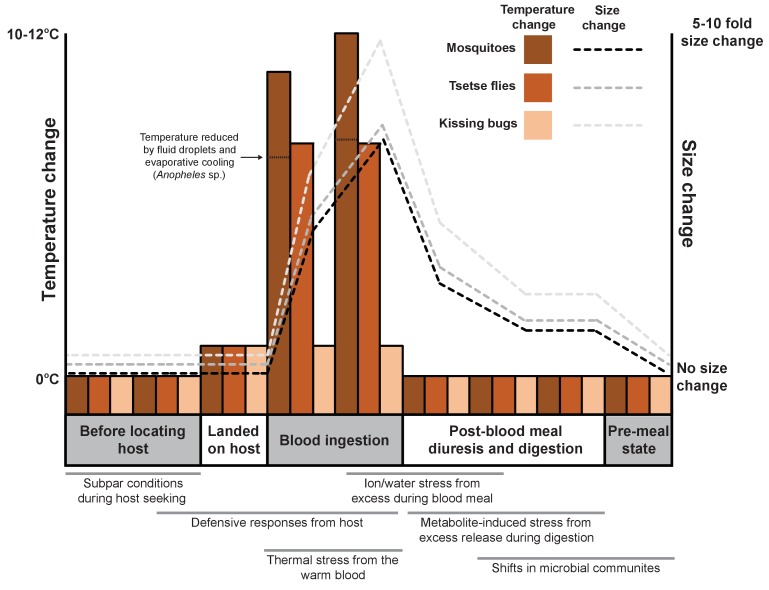
Feeding on blood leads to various physiological shifts in arthropods. Changes in body temperature (bars; left axis) and size (dashed lines; right axis) for three species of arthropod (mosquitoes, tsetse flies and kissing bugs) before, during and after feeding on the blood of a vertebrate. The process of blood feeding exposes the arthropods to a number of stresses that are likely to necessitate a biological response (shown under the graph; gray bars indicate when each stress is likely to occur).

The heat of the vertebrate represents a frequently overlooked stress associated with blood ingestion even though the temperature of blood-feeding arthropods may increase by up to 15°C in less than one minute during their meal (Benoit et al., 2011; Lahondère and Lazzari, 2012; Lahondère and Lazzari, 2015). It is known that the thermal stress generated by the blood meal can trigger the arthropod’s heat shock response, as demonstrated by the increased production of heat shock proteins (Benoit et al., 2011).

Arthropods use a range of different mechanisms to reduce heat stress during blood feeding: some open small holes called spiracles on their surface to increase heat loss from breathing; the mosquito *Anopheles stephensi* (Lahondère and Lazzari, 2012) retains drops of urine on the abdomen that cool as they evaporate; and tsetse flies cool their meals by feeding from pools of blood instead of directly from blood vessels (Lahondère and Lazzari, 2015). Now, in eLife, Claudio Lazzari from the University of Tours and colleagues – including Chloé Lahondère as first author – report a heat exchange mechanism that enables kissing bugs to control their temperature as they ingest a warm blood meal (Lahondère et al., 2017).

Heat dissipates quickly from the head of a kissing bug while it feeds, allowing the rest of the body to remain at ambient temperature. This is in stark contrast to what happens in many of the other blood-feeding arthropods examined by Lahondère et al. (most of the body increases to a temperature near that of the host (Lahondère and Lazzari, 2012; Lahondère and Lazzari, 2015).

Using histology, micro-computed tomography and X-ray synchrotron imaging, Lahondère et al. – who are based at Tours, the Universidade Federal de Minas Gerais, the University of Saskatchewan and the Canadian Light Source Inc. – noted the close proximity of the circulatory and ingestion systems in the head. They suggested that the flow of the kissing bug’s blood toward the head could help to cool the blood meal as it moves through the ingestion system, before it reaches the thorax and abdomen. This is an example of a countercurrent heat exchanger – a system where two fluids that flow in opposite directions act to reduce the temperature difference between them.

To test this hypothesis Lahondère et al. conducted a set of experiments in which they stopped blood circulating around the kissing bug by severing the dorsal vessel. This intervention caused the abdominal temperature of the bug to soar to near that of the host. This triggered the production of more heat shock proteins in the bug, and demonstrates that the head thermal exchanger plays a critical role in dissipating heat from the blood meal.

When an arthropod ingests a blood meal, there are a multitude of stresses that must be prevented or tolerated. As well as heat stress, arthropods must eliminate large amounts of excess water and ions (Beyenbach and Piermarini, 2011), detoxify the harmful products that result from a high-protein diet (Sterkel et al., 2017), and tolerate massive increases in the number of bacteria in their gut (Wang et al., 2011). By improving our understanding of the protective mechanisms used by blood-feeding arthropods to counter bouts of blood meal-induced stress, the work of Lahondère et al. will help with efforts to develop new ways to prevent blood feeding and reduce the spread of diseases carried by blood-feeding arthropods.
